# Informal Status and Taking Charge: The Different Roles of OBSE, P-J Fit, and P-S Fit

**DOI:** 10.3389/fpsyg.2020.01994

**Published:** 2020-08-14

**Authors:** Chuanjun Deng, Shudi Liao, Zhiqiang Liu, Yucheng Zhang, Yan Bao

**Affiliations:** ^1^Business School, Henan University, Kaifeng, China; ^2^Business School, Hubei University, Wuhan, China; ^3^Hubei Center for Studies of Human Capital Development Strategy and Policy, and Key Research Base of Humanities and Social Science of Hubei Province, Wuhan, China; ^4^School of Management, Huazhong University of Science and Technology, Wuhan, China; ^5^School of Economics and Management, Hebei University of Technology, Tianjin, China; ^6^School of Management, Xi’an Jiaotong University, Xi’an, China

**Keywords:** knowledge workers’ informal status, taking charge, organization-based self-esteem, person-job fit, person-supervisor fit

## Abstract

Status in an organization is considered a significant antecedent to an employee’s work-related behaviors. However, the relationship between knowledge workers’ informal status and “taking charge” has been ignored in previous human resource management research. Based on the self-consistency theory, this study examines the mechanisms underlying the influence of knowledge workers’ informal status on taking charge. Data were collected from 337 dyads of employees and their immediate supervisors in 24 enterprises and companies. The results of moderated-mediation analysis indicate organization-based self-esteem (OBSE) fully mediated the positive relationship between knowledge workers’ informal status and taking charge, whereas person-job fit (P-J fit) and person-supervisor fit (P-S fit) each moderated the relationship between knowledge workers’ informal status and OBSE, in addition to the indirect effect of knowledge workers’ informal status on taking charge. Specifically, the indirect effect was strongest when P-J fit or P-S fit was high. The theoretical and managerial implications of the findings, limitations of the study, and future research directions are discussed.

## Introduction

Status of knowledge workers has been a key issue in human resource management (HRM) research for decades (Bunderson, 2003; Heyden et al., 2018). There are two types of status in the workplace, including informal and formal status (Anderson et al., 2012, 2015). Knowledge workers’ informal status is defined as respect, admiration, and voluntary deference conferred by others. Knowledge workers’ formal status is reflected in one’s job level, titles, and positions, which are conferred by the legitimacy of HRM systems. In contrast, knowledge workers’ informal status is obtained from social interactions within the knowledge team. Although formal status stems from official HRM recognition, knowledge workers’ informal status reflects employees’ competency and hard power. Moreover, with increased environmental uncertainty, HRM systems have come to recognize the difficulty of relying only on macrolevel reforms (Morrison and Phelps, 1999; Kim et al., 2015). It is therefore urgently necessary for HRM systems to supplement microlevel changes to improve its flexibility and adaptability. Previous HRM research of knowledge worker has generally been limited to the relationship between status and innovation (Ibarra, 1993; Perrett and Negro, 2006; Cattani and Ferriani, 2008; Duguid and Goncalo, 2015; Sahib, 2015), helping behavior (Agneessens and Wittek, 2012; Brandts et al., 2015), and unethical behaviors (Charness et al., 2013; Edelman and Larkin, 2014). Until now, little research has examined the relationship between knowledge workers’ status and “taking charge” behavior.

An employee takes charge through his/her voluntary and constructive efforts to promote organizational change in how tasks are carried out at various levels, from individual jobs to departments, work units, and whole organizations (Morrison and Phelps, 1999). Taking charge is crucial for both individual and organizational success in the uncertain environment of modern society. When an employee is voluntarily taking charge, his/her active and constructive efforts facilitate functional change and enhance management effectiveness (Kim et al., 2015). Accordingly, employees who take charge may be regarded as showing a form of leadership (Morrison and Phelps, 1999). Taking charge includes raising, promoting, and implementing new ideas and identifying, promoting, and implementing opportunities for change, which is thus more transformative than individual innovative behaviors (Morrison and Phelps, 1999). Therefore, taking charge not only may result in positive effects but also can create impression risk and relationship conflict on account of its challenging nature (Morrison and Phelps, 1999; McAllister et al., 2007).

Who will be more likely to take charge? Anderson and Kilduff (2009) found that high-informal-status employees were likely to develop solutions before others and that their ideas were ultimately adopted by their organizations more than 94% of the time compared with others. Bales et al. (1951) argued that high-informal-status members’ voice challenges are 15 times more frequent than those of low-status members and about 5 times more frequent than those of middling-status employees in the working group. Successfully taking charge largely depends on employees’ power within the organization (Morrison and Phelps, 1999). Therefore, we suggest that knowledge workers’ informal status is more likely than formal status to be the antecedent of taking charge. According to the self-consistency theory (Korman, 1970), as organization-based self-esteem (OBSE) increases, an individual’s self-perceptions and behaviors improve. In particular, to the extent that informal status influences employees’ self-perceptions and OBSE, employees become more likely to improve their behavior such that it aligns with their self-perceptions.

Employees have a strong need to interact with their work environments (Heyden et al., 2018). Their experiences at workplace depends on the “fit” with supervisors, job roles, and other organizational attributes (Schneider, 1987; Ng and Feldman, 2010). Supervisors provide high support and power to subordinates whose values, personalities, and ways of thinking are consistent with their own. Employees prefer jobs that match their abilities and needs, such that their capabilities and values can be fully realized. We therefore suggest that person-job fit (P-J fit) and person-supervisor fit (P-S fit) have a significant influence on the relationships between knowledge workers’ informal status, OBSE, and taking charge, respectively. The effects of each type of fit are different (Boon and Biron, 2016). Self-consistency theory suggests that when employees have high person-environment fit (P-E fit), they are perceptive to changes in social comments and quickly adjust their self-evaluations and behaviors such that social comments, self-evaluations, and behavioral choices are consistent (Korman, 1970). In other words, P-J fit and P-S fit affects employees’ relative informal status, which in turn ensures that individuals’ self-evaluations and behaviors align with their status; their OBSE and willingness to take charge therefore also change over time.

This study makes four theoretical contributions. First, this study uncovered the relationship between knowledge workers’ informal status and taking charge and added to the literature that explores the antecedents of employee taking charge. Second, based on the self-consistency theory, this study improves our understanding of OBSE as a mediator to explain the relationship between knowledge workers’ informal status and taking charge. Although the role of OBSE as a mediator has been widely discussed in other theoretical domains, it has not been considered in research on status and taking charge. In this study, OBSE explains the relationship between informal status and knowledge workers’ willingness to take charge, which further enriches the theoretical content of the OBSE literature. Third, the study outlines the moderators of the self-consistency theory process (i.e., P-J fit, and P-S fit). A lack of research on these moderators has thus far restricted our understanding of the boundary conditions for enhancing the effects of knowledge workers’ informal status. Finally, the findings of this study’s conclusions provide important practical insights for organizations, helping them to improve knowledge workers’ willingness to take charge and their organizational adaptability.

## Hypotheses

### Informal Status and Taking Charge

According to the self-consistency theory, people are motivated to behave in ways that fit with others’ comments for maintaining cognitive consistency between subjective attitudes and behaviors (Korman, 1970). Employees’ behavioral responses are motivated by a desire to be consistent with the comments of others. There are two ways that this motivation translates into taking charge. First, knowledge workers with high levels of informal status have a greater impetus to carry out tasks beyond the regular responsibilities of their position. For instance, Howell et al. (2015) showed that higher-informal-status employees take on a wider range of responsibilities to maintain their self-image. Second, leaders and colleagues are more likely to delegate and grant job autonomy to employees with higher levels of informal status, which in turn develops their ability to taking charge. For instance, prior research has found that high-informal-status employees not only have more autonomy at work, but also their proposed solutions and suggestions for changes are more easily adopted by organizations and work teams (Anderson and Kilduff, 2009). Additionally, research has shown that high-informal-status employees receive more trust, cooperation, and support from their teammates (De Kwaadsteniet and Van Dijk, 2010) and are better at discovering problems and deficiencies in the work environment (Anderson and Kilduff, 2009; Anderson et al., 2012). Therefore, knowledge workers with high informal status can solve problems in organizations more efficiently than their low-status colleagues. Based on the above analysis, we put forth the following hypothesis:

*Hypothesis 1:* Knowledge workers’ informal status is positively related to taking charge.

### Knowledge Workers’ Informal Status and OBSE

Knowledge workers’ informal status in an organization results from social evaluations and yields consistent self-evaluations. Self-consistency theory (Korman, 1970) suggests that employees strive to align their self-cognition with their sense of how others perceive them. Informal status indicates personal capability and social value because it is conferred by colleagues or teammates within an organization based on ability, contribution, and influence. OBSE refers to employees’ perception of themselves as valued, effectual, and worthwhile in their work units (Pierce et al., 1989). Based on the status–function theory, high-informal-status knowledge workers enjoy more social capital and earn more respect, social support, happiness, and resources in the knowledge team (Anderson et al.; Chen et al., 2012). This is because high-informal-status individuals often have positive self-evaluations (Thye, 2000). Specifically, in comparison with their low-status teammates, high-status employees are more likely to be chosen as partners and cooperators. Besides, those members tend to receive the most requests for help or to serve as role models (Anderson et al., 2015). Positive social evaluations of an individual by others lead to positive self-evaluations. Following the status attribution perspective (Iatridis and Fousiani, 2009), peers in the team are more likely to attribute the success of high-status individuals to their exceptional capabilities, whereas low-status colleagues are seen as lacking abilities. Additionally, Pierce and Gardner (2004) argued that individual career success is the backbone of employees’ OBSE. Together, these theoretical propositions and empirical findings suggest that knowledge workers maintain a self-evaluation consistent with their status level in the knowledge team. Therefore, we propose the following hypothesis:

*Hypothesis 2:* Knowledge workers’ informal status is positively related to OBSE.

### The Mediating Effect of OBSE

Self-consistency theory (Korman, 1970) suggests that people try to behave consistently with their self-perceptions. Specifically, knowledge workers’ informal status influences their self-evaluation, which in turn affects their willingness to taking charge. According to the definition of informal status, high-informal-status knowledge workers are valued more in organizations and thus enjoy respect, empowerment, and esteem than low-informal-status members (Lynn et al., 2009). This encourages high-informal-status knowledge workers to maintain self-cognition and behavioral choices consistent with their status. Informal status contributes to knowledge workers’ ability to realize social value, which makes them more willing to actively taking charge. For instance, research based on the theory of status characteristics shows that high-informal-status individuals are more likely to act as pioneers and make contributions to their teams because these activities are conducive to winning followers and effectively maintaining a high-status image (Simpson et al., 2012). Consequently, high-status members will choose to engage in behaviors beyond their official roles, including taking charge and helping others. Also, the more positive self-evaluations of high-informal-status knowledge workers prompt them to develop taking charge more actively. Informal status is a synonym for individual competence; when this is reflected in employees, it increases self-esteem and leads to consistent individual behaviors. For instance, research has shown that knowledge workers’ informal status reflects individual competence and encourages members to take risks for the success of the organization and voluntarily contribute to their units (Willer, 2009). The most effective way for a member to maintain his/her positive image is to show competence and generosity (Anderson and Kilduff, 2009). Taking charge not only shows generosity but also reveals competence to the organization. This tendency is reinforced by the self-consistency motivation, such that behaviors reflect self-perceptions to avoid cognitive dissonance (Korman, 1970). Therefore, we propose the following hypothesis:

*Hypothesis 3:* OBSE mediates the relationship between knowledge workers’ informal status and taking charge.

### The Moderating Effects of P-J fit and P-S Fit

Person-job fit refers to the extent to which an employee’s skills, knowledge, and abilities adhere to the requirements of a job or task (Boon and Biron, 2016). Edwards (1991) separated P-J fit into two distinct parts: the demands-abilities fit, which describes the alignment of a member’s ability, knowledge, and skills with the demands of a job, and the needs-supplies fit, which describes how well what an employee needs to be satisfied at work aligns with what the employer provides. Park et al. (2011) suggested that employees who match well with their jobs will be more successful in their performance. Therefore, high P-J fit can improve an employee’s relative status within a group.

Person-job fit can significantly moderate the relationship between knowledge workers’ informal status and OBSE. Self-consistency theory holds that, to maintain cognitive consistency, individuals are motivated to keep their self-evaluations aligned with social perceptions (Korman, 1970). Specifically, employees with high levels of P-J fit are more likely to report higher levels of performance and reward at work (Cable and DeRue, 2002) and feel that they have more opportunities to take part in decision-making (Boon and Biron, 2016). Sekiguchi and Huber (2011) also found that employees with high P-J fit are more likely to hold knowledge-intensive positions. In these cases, employees with high P-J fit perceive themselves as having achieved higher relative informal status than other members. Therefore, their OBSE is improved because their relative status has increased. We thus propose the following hypothesis:

*Hypothesis 4a:* P-J fit moderates the relationship between knowledge workers’ informal status and OBSE such that the relationship is stronger when P-J fit is high rather than low.

From the perspective of self-categorization, P-S fit enhances members’ identity recognition and improves OBSE. P-S fit refers to the match between a subordinate and his/her supervisor’s characteristics, including personality, values, and behavioral styles (Chuang and Shen, 2007). Specifically, individuals, regardless of their work role as supervisors or subordinates, prefer to share knowledge and information with others that are similar to themselves (Hwang et al., 2015; Heyden et al., 2018). This self-generalization further improves employees’ OBSE. However, if there is a mismatch between an employee and his/her supervisors or team members, then they will suffer workplace ostracism and isolation (Quade et al., 2017), which can negatively impact OBSE. Based on the social classification, people continuously develop and maintain their sense of self-esteem by comparing themselves with others and aligning themselves with favorable subgroups (Tajfel and Turner, 1986). Specifically, when P-S fit is high, employees align themselves with higher-status subgroups and identify their relative status as similar to that of their superiors or categorize themselves as future high-status candidates (DiBenigno and Kellogg, 2014). As a result, their OBSE will increase significantly.

According to similarity–attraction theory, people tend to develop positive social relationships with similar others, especially when interactions with similar supervisors or other high-status members result in status “leakage” (Graffin et al., 2008). The similarity–attraction model shows that appraisers have self-schemas for performance evaluation and social relationships. These reinforce not only positive self-images but also a person’s preference for similar others. Therefore, OBSE increases significantly because of this positive psychological cue. For instance, in a role-playing experiment (Eagleson et al., 2000), subjects acting as managers preferred to select similar members for the center position of a network, which in turn led to relative status changes among the network members. The participants selected to be the network center experienced a significant increase in OBSE, whereas that of others decreased. Moreover, a strong P-S fit results in increasing momentum in the form of opportunities for vocational training and to take on more important tasks (Thompson et al., 2006). Studies have found that a match in personalities between supervisors and employees can predict subordinates’ future promotions (Schaubroeck and Lam, 2002). Every member of a work team experiences the process of attraction–selection–attrition among supervisors and subordinates. If employees ultimately fit well with their supervisors and become insiders, then their value will be reflected in other members, which increases their relative status. OBSE, in turn, is significantly improved because of those members perceiving themselves as having a higher relative status due to the good P-S fit. Based on the above analysis, we propose the following hypothesis:

*Hypothesis 4b:* P-S fit moderates the relationship between knowledge workers’ informal status and OBSE such that the relationship is stronger when P-S fit is high rather than low.

Thus far, our analysis has developed theoretical underpinnings for the mediating effect of OBSE between knowledge workers’ informal status and taking charge, and the moderating effects of P-J and P-S fits on the relationship between knowledge workers’ informal status and taking charge, respectively. Employees with low levels of OBSE are less likely to take charge, and those with high P-J fit and P-S fit are more likely to experience an increase in OBSE when they perceive themselves to have higher relative informal status. Therefore, the theoretical rationales supporting the previous hypotheses suggest a moderated-mediation model (Hayes and Scharkow, 2013). We thus propose the following hypotheses:

*Hypothesis 5a:* P-J fit moderates the indirect effect of knowledge workers’ informal status on taking charge via OBSE, such that the indirect effect is strongest when P-J fit is high.
*Hypothesis 5b:* P-S fit moderates the indirect effect of knowledge workers’ informal status on taking charge via OBSE, such that the indirect effect is strongest when P-S fit is high.

**FIGURE 1:**
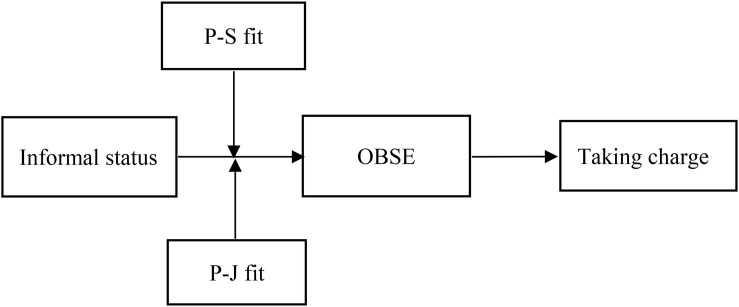
Research framework.

Figure 1 presents the study’s theoretical framework.

## Materials and Methods

### Sample and Procedures

This study explores the relationships between knowledge workers’ informal status, P-J fit, P-S fit, OBSE, and taking charge in a knowledge-intensive context. Data were collected from 500 knowledge works and their supervisor from organizations in knowledge-intensive industries in China, such as the pharmaceutical, machinery, and energy industries. To avoid the issue of common method variance, dyadic questionnaires were distributed, including employees and their supervisors. Subordinates were invited to fill out the Employee Questionnaire in which they reported their OBSE and taking charge. The direct supervisor rated the members’ informal status, P-J fit, and P-S fit. The Employee Questionnaire and Leader Questionnaire were matched by a random three-digit code. Participation was voluntary and anonymous. In total, 500 supervisor–subordinate dyadic questionnaires were sent to managers and their employees, and 324 valid dyadic questionnaires were returned in 91 teams, yielding a response rate of 64.80%.

In the final sample, 52.80% of the followers were male. The average tenure of participants was 5.06 (s.d. = 5.24) years, and an average age of participants was 30.16 (s.d. = 5.96) years. A total of 71.50% of the subordinates had bachelor degrees or greater, and 94.10% of the members were working in private enterprises. To the position hierarchy of the subordinates, the high, middle, and low levels were 2.40, 25.00, and 72.60%, respectively. All the employees had worked with their supervisors for at least half a year, and the average time of subordinate–supervisor working relation was 3.02 years.

### Measures

The scales were initially in English; we followed the back-translation principle of Brislin (1986), and all the surveys were translated into Chinese and then into English to make sure the version is consistent.

#### Knowledge Workers’ Informal Status

Knowledge workers’ informal status was evaluated by their supervisors. A five-item scale adopted from Anderson et al. (2008) and Spataro (2012) was used to capture knowledge workers’ informal status in the organization. A sample item is “The organization values the contributions of this knowledge worker.” The participants responded on a 7-point Likert scale, ranging from 1 (strongly disagree) to 7 (strongly agree). Cronbach α was 0.87.

#### Taking Charge

Knowledge workers’ willingness to take charge was self-reported. A 10-item scale developed by Morrison and Phelps (1999) was applied to measure employees’ willingness to take charge. A sample item is “I frequently try to bring about improved procedures for the work unit or department.” The participants responded on a 7-point Likert scale ranging from 1 (strongly disagree) to 7 (strongly agree). Cronbach α was 0.92.

#### OBSE

Organization-based self-esteem was self-reported by employees. An eight-item scale was used to measure employees’ OBSE (Pierce et al., 1989). A sample item is “I am an important part of this organization.” The participants responded on a 7-point Likert scale, ranging from 1 (strongly disagree) to 7 (strongly agree). Cronbach α was 0.86.

#### P-J Fit

Person-job fit fit was evaluated by the supervisors. A three-item scale was used to measure employees’ P-J fit (Cable and DeRue, 2002). A sample item is “Fit between the employee’s knowledge and skills and the job requirements.” The participants responded on a 7-point Likert scale, ranging from 1 (strongly disagree) to 7 (strongly agree). Cronbach α was 0.83.

#### P-S Fit

Person-supervisor fit was evaluated by the supervisors. A three-item scale was used to measure employees’ P-S fit (Chuang and Shen, 2007). A sample item is “the employee is similar to me in analyzing problems.” The participants responded on a 7-point Likert scale, ranging from 1 (strongly disagree) to 7 (strongly agree). Cronbach α was 0.86.

#### Control Variables

Based on the recommendations of previous research (Anicich et al., 2015; Li et al., 2017), the variables included gender (1 = male, 0 = female), age, education level, tenure, industry, and unit nature. We also controlled for formal status recognized by HRM system integrating methods adapted from the ways developed by Aquino et al. (1999) and Yuan and Woodman (2010) to measure the Gini coefficient of the formal status in an organization.

## Results

To ascertain that our six measures were distinct constructs, we conducted five confirmatory factor analyses to compare the five-factor model with alternative models: one-, two-, three-, and four-factor models, respectively. Table 1 shows that the five-factor model (χ^2^/*df* = 2.04, root mean square error of approximation = 0.06, goodness-of-fit index = 0.91, incremental fit index = 0.93, and comparative fit index = 0.93) displayed a better match to the data than the alternative models. Therefore, the results supported our treating the variables as measuring distinct constructs.

**TABLE 1:** Confirmatory factor analyses.

	χ^2^	*df*	χ^2^/*df*	RMSEA	GFI	IFI	CFI
(1) Five-factor model (knowledge workers’ informal status/P-J fit/P-S fit/OBSE/taking charge)	750.33	367	2.04	0.06	0.91	0.93	0.93
(2) Four-factor model (knowledge workers’ informal status/P-J fit/P-S fit/OBSE + taking charge)	1,187.36	371	3.20	0.08	0.83	0.85	0.85
(3) Three-factor model (knowledge workers’ informal status/P-J fit/P-S fit + OBSE + taking charge)	1,645.85	374	4.40	0.10	0.75	0.77	0.77
(4) Two-factor model (knowledge workers’ informal status, P-J fit + P-S fit + OBSE + taking charge)	2,246.28	376	5.97	0.13	0.62	0.66	0.66
(5) One-factor model (knowledge workers’ informal status + P-J fit + P-S fit + OBSE + taking charge)	3,438.53	377	9.12	0.16	0.43	0.45	0.44

*CFI, comparative fit index; GFI, goodness-of-fit index; IFI, incremental fit index; RMSEA, root mean square error of approximation.*

Table 2 reports the means, standard deviations, and correlations of the variables. All of the reliability estimates were acceptable (i.e., α > 0.70).

**TABLE 2:** Means, standard deviation, and correlations (*N* = 324).

	M	SD	1	2	3	4	5	6	7	8	9	10	11	12
(1) Gender	0.49	0.17	–											
(2) Age	30.16	5.96	−0.13*	–										
(3) Education	2.72	0.67	0.01	–0.10	–									
(4) Tenure	5.06	5.24	0.01	0.70**	–0.10	–								
(5) Industry	6.31	4.84	0.09	–0.03	−0.18**	0.01	–							
(6) Unit nature	2.94	1.80	–0.07	–0.09	−0.16**	−0.20**	0.01	–						
(7) Formal status	0.40	0.21	−0.13*	0.14*	0.17**	0.02	−0.11*	0.09	–					
(8) Taking charge	4.53	0.77	−0.16**	0.06	0.10	0.10	–0.09	−0.20**	–0.02	(0.92)				
(9) OBSE	4.67	0.67	–0.11	0.01	–0.00	0.05	0.07	−0.18**	0.03	0.55**	(0.86)			
(10) P-S fit	4.27	1.12	–0.02	0.07	0.10	–0.01	–0.07	–0.04	0.11	0.21**	0.24**	(0.86)		
(11) P-J fit	4.94	1.02	0.01	0.05	–0.01	0.06	–0.04	–0.06	–0.01	0.25**	0.31**	0.51**	(0.83)	
(12) Informal status	4.42	1.04	–0.01	0.15**	–0.00	0.11*	0.02	–0.02	0.00	0.23**	0.26**	0.52**	0.64**	(0.87)

*p < 0.05, **p < 0.01, ***p < 0.001 (two-tailed).

Hypotheses 1 and 2 predicted that knowledge workers’ informal status is positively related to taking charge and OBSE. We used hierarchical multiple regression analysis to test these hypotheses. The results in Table 3 for Models 2 and 4 show that knowledge workers’ informal status was positively related to taking charge (β = 0.18, *p* < 0.001) and OBSE (β = 0.19, *p* < 0.001). Therefore, Hypotheses 1 and 2 were supported.

**TABLE 3:** Results of hierarchical regression analysis (*N* = 324).

	Taking charge				OBSE				Taking charge		OBSE		Taking charge			
					
	M1		M2		M3		M4		M5		M6		M7		M8	
								
	β	SE	β	SE	β	SE	β	SE	β	SE	β	SE	β	SE	β	SE
(Constant)	5.18***	0.38	5.33***	0.38	5.27***	0.34	5.43***	0.33	4.91***	0.33	5.33***	0.32	5.24***	0.36	4.92***	0.33
Gender	−0.27**	0.09	−0.27***	0.08	−0.18*	0.08	−0.18*	0.07	−0.17*	0.07	−0.13**	0.07	−0.21**	0.08	−0.15*	0.07
Age	–0.01	0.01	–0.01	-0.01	–0.01	0.01	–0.01	0.01	–0.00	0.01	–0.01	0.01	–0.01	0.01	–0.01	0.01
Education	0.08	0.07	0.07	0.06	–0.03	0.06	–0.04	0.06	0.09	0.05	–0.08	0.05	0.03	0.06	0.06	0.06
Tenure	0.02	0.01	0.02	0.01	0.01	0.01	0.01	0.01	0.01	0.01	0.00	0.01	0.01	0.01	0.01	0.01
Industry	–0.01	0.01	–0.01	0.01	0.01	0.01	0.01	0.02	–0.02	0.01	0.01	0.01	–0.01	0.01	−0.02*	0.02
Unit nature	0.08**	0.02	−0.08***	0.02	−0.08***	0.02	−0.07***	0.02	–0.03	0.02	−0.07***	0.02	−0.07**	0.02	–0.03	0.02
Formal status	–0.16	0.21	–0.14	0.21	0.17	0.19	0.18	0.18	–0.25	0.20	0.20	0.17	–0.11	0.20	–0.22	0.18
Informal status			0.18***	0.04			0.19***	0.04	0.07	0.04	0.08	0.05	0.10	0.06	0.06	0.05
OBSE									0.42***	0.04					0.37***	0.04
P-J fit											0.21***	0.05	0.18**	0.06	0.08	0.05
P-S fit											0.06	0.04	0.06	0.05	0.03	0.04
Informal status × P-J fit											0.14**	0.05	0.15**	0.05	0.08	0.05
Informal status × P-S fit											0.09*	0.05	0.11*	0.05	0.06	0.05
*R*^2^	0.09		0.13		0.06		0.13		0.36		0.24		0.23		0.38	
AR^2^	0.07		0.11		0.04		0.11		0.34		0.21		0.20		0.36	
F	4.59***		4.23***		2.86**		5.80***		19.54***		8.27***		7.65***		14.66***	

*p < 0.05, **p < 0.01, ***p < 0.001 (two-tailed).

Hypothesis 3 predicted that OBSE mediates the relationship between knowledge workers’ informal status and taking charge. The results in Table 3 for Model 5 show that the relationship between knowledge workers’ informal status and taking charge was mediated by OBSE (β = 0.42, *p* < 0.001). Specifically, after incorporating OBSE into the third hierarchy, the previous relationship between knowledge workers’ informal status and taking charge changed from 0.18 (*p* < 0.001) to 0.07 [*p* > 0.05, not statistically significant (n.s.)]. Therefore, Hypothesis 3 was supported. Additionally, bootstrapping 5,000 samples indicated that OBSE had a significant mediation effect on the relationship between knowledge workers’ informal status and taking charge. The indirect effect was 0.10 at a 95% confidence interval (CI) = 0.06–0.15. Thus, Hypothesis 3 was further supported.

**FIGURE 2:**
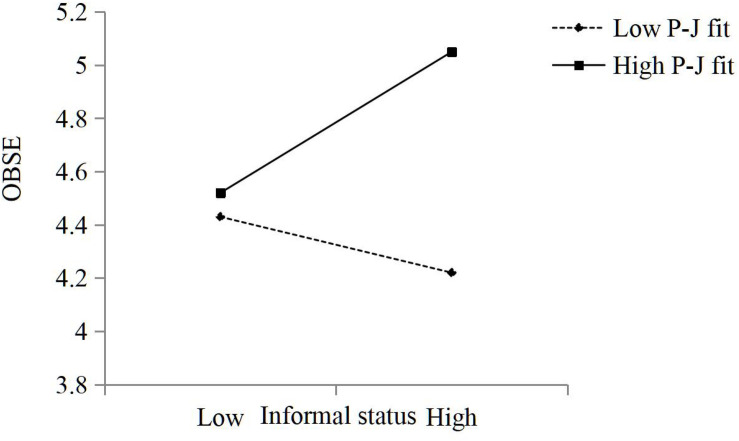
The moderating role of P-J fit in the relationship between knowledge workers’ informal status and OBSE.

Hypothesis 4a suggested that P-J fit moderates the relationship between knowledge workers’ informal status and OBSE such that the relationship is stronger when P-J fit is high rather than low. The results in Table 3 for Model 6 show that the coefficient for the interaction between knowledge workers’ informal status and OBSE was significant and positive (β = 0.14, *p* < 0.01). The results were plotted for P-J fit values corresponding to 1 s.d. below and above the mean (Figure 2). A simple slopes test showed that the slope for low P-J fit was significant (β = −0.15, *p* < 0.10), and the slope for high P-J fit was positive and significant (β = 0.40, *p* < 0.001). Thus, Hypothesis 4a is supported.

**FIGURE 3:**
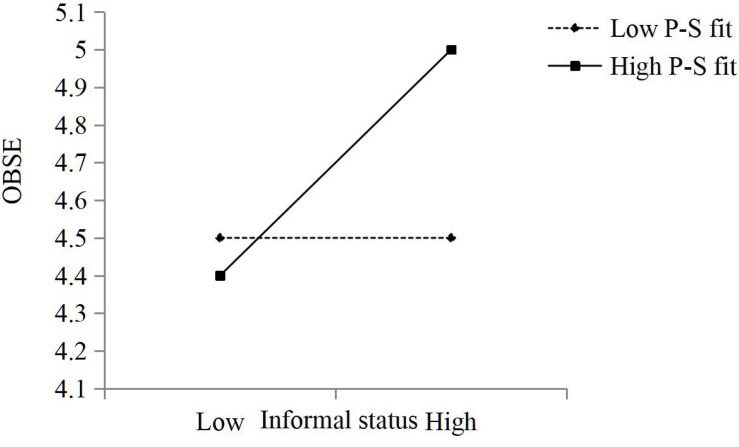
The moderating role of P-S fit in the relationship between knowledge workers’ informal status and OBSE.

Hypothesis 4b predicted that P-S fit moderates the relationship between knowledge workers’ informal status and OBSE such that the relationship is stronger when P-S fit is high rather than low. The results in Table 3 for Model 6 show that the coefficient for the interaction between knowledge workers’ informal status and OBSE was significant and positive (β = 0.09, *p* < 0.05). The results were plotted for P-S fit values corresponding to 1 s.d. below and above the mean (Figure 3). A simple slopes test showed that the slope for low P-S fit was non-significant (β = 0.00, *p* > 0.05, n.s.), and the slope for high P-S fit was positive and significant (β = 0.42, *p* < 0.001). Thus, Hypothesis 4b was supported.

Hypotheses 5a and 5b predicted that P-J fit and P-S fit independently moderate the indirect effect of knowledge workers’ informal status on taking charge via OBSE, such that the indirect effect is strongest when P-J or P-S fit is high rather than low. To empirically test the moderated-mediation hypotheses, we followed the procedure of Preacher et al. (2007) to compare the conditional indirect effects, and the moderated mediation arises when the strength of the mediated relationship is affected by the level of a moderator (Edwards and Lambert, 2007). The results of the conditional indirect effects showed that the indirect effects of informal status on taking charge via OBSE were significantly increased when the P-J or P-S fit is high. Meanwhile, under low P-J or P-S fit, the same indirect effect decreased insignificantly. Specifically, the results in Table 4 show that OBSE had a significant mediation effect on the relationship between knowledge workers’ informal status and taking charge when P-J fit was high (i.e., conditional mediation effect = 0.15, 95% CI = 0.09–0.22, statistically significant) versus low (i.e., conditional mediation effect = −0.05; 95% CI = −0.11 to 0.01, n.s.). And the results in Table 5 show that OBSE had a significant mediation effect on the relationship between knowledge workers’ informal status and taking charge when P-S fit was high (i.e., conditional mediation effect = 0.16, 95% CI = 0.11–0.23, statistically significant) versus low (i.e., conditional mediation effect = −0.00; 95% CI = −0.05–0.05, n.s.). Thus, Hypotheses 5a and 5b were supported.

**TABLE 4:** Conditional indirect effect(s) at values of the moderator of P-J fit.

Taking charge								

Mediator	Index	SE	Boot LL 95% CI	Boot UL 95% CI	P-J fit	Effect	Boot LL 95% CI	Boot UL 95% CI
OBSE	0.10	0.02	0.07	0.14	Low P-J fit (−1 s.d.)	−0.05	−0.11	0.01
					Middle P-J fit (at mean)	0.05	0.01	0.10
					High P-J fit (+1 s.d.)	0.15	0.09	0.22

*Values for quantitative moderators are −1 s.d., at mean, and +1 s.d.; bootstrap sample size = 5,000. LL, lower limit; UL, upper limit; CI, confidence interval.*

**TABLE 5:** Conditional indirect effect(s) at values of the moderator of P-S fit.

Taking charge								

Mediator	Index	SE	Boot LL 95% CI	Boot UL 95% CI	P-S fit	Effect	Boot LL 95% CI	Boot UL 95% CI
OBSE	0.07	0.02	0.04	0.11	Low P-S fit (−1 s.d.)	−0.00	−0.05	0.05
					Middle P-S fit (at mean)	0.08	0.04	0.13
					High P-S fit (+ 1 s.d.)	0.16	0.11	0.23

*Values for quantitative moderators are −1 s.d., at mean, and + 1 s.d.; bootstrap sample size = 5,000. LL, lower limit; UL, upper limit; CI, confidence interval.*

## Discussion

Although growing attention has been paid to status and taking charge in the HRM literature, it is surprisingly rare for HRM research to uncover the mechanisms by which knowledge workers’ informal status affects employees’ taking charge. To address this gap, we developed and empirically examined the process of how knowledge workers’ informal status influences their taking charge. Specifically, our findings show that there is a positive relationship between knowledge workers’ informal status and taking charge and that this relationship is fully mediated by OBSE. P-J fit and P-S fit both moderate the relationship between knowledge workers’ informal status and OBSE, respectively. Specifically, P-J fit and P-S fit independently moderate the relationship between knowledge workers’ informal status and OBSE, such that the relationship is stronger when the fit is high rather than low. Furthermore, the two types of fit moderated the indirect effect of knowledge workers’ informal status on taking charge via OBSE, such that the indirect effect is strongest when the fit is high.

### Theoretical Implications

These results have several implications for research. First, based on the self-consistency framework, this study constructed and systematically analyzed a contingency model of the relationship between knowledge workers’ informal status and taking charge and thus contributes to the theories of status and taking charge. Status and its effects have been of central concern to HRM research for several decades. However, no research has addressed how informal status influences knowledge workers’ taking charge. Prior studies have explored the relationships between informal status and innovation behavior (Perrett and Negro, 2006; Cattani and Ferriani, 2008; Duguid and Goncalo, 2015; Sahib, 2015), helping behavior (Agneessens and Wittek, 2012; Brandts et al., 2015), organizational citizenship behavior (Aquino and Bommer, 2003; Marr and Thau, 2014), and unethical behaviors (Charness et al., 2013; Edelman and Larkin, 2014). This study thus expands the literature by empirically testing the relationships between knowledge workers’ informal status and taking charge.

Second, this study empirically tested and validated the mediating role that OBSE plays in the relationship between knowledge workers’ informal status and taking charge. In addition, our findings indicate that P-J and P-S fits independently moderated the indirect effect of knowledge workers’ informal status on taking charge via OBSE. The results therefore expand the theoretical content of OBSE. Pierce and Gardner (2004) argued that while OBSE has been studied as a mediator in a diverse range of contexts (e.g., in the relationships between job characteristics and citizenship behavior, perceived organizational support and job performance, and leader–member exchange and contextual performance), further HRM research is needed to understand the precise mechanisms by which this mediation takes place. Our study heeded their recommendation to construct a research framework with a solid theoretical foundation. We systematically examined OBSE’s mediating effects and thus confirmed its role in employees’ knowledge workers’ informal status and willingness to taking charge, thereby expanding the theoretical scope of OBSE theory.

Third, this study confirms that P-E fit is key to the influence of knowledge workers’ informal status on taking charge, making great contributions to research on P-E fit theory, as follows. The study is the first to incorporate the effects of P-E interaction into a research framework of status, yielding novel findings. The results show that P-J and P-S fits are important boundary conditions of the relationship between knowledge workers’ informal status and OBSE. This study shows that P-J and P-S fits each moderate the indirect effect of knowledge workers’ informal status on taking charge via OBSE, such that the indirect effect is strongest when P-J fit or P-S fit is high. Previous studies have generally failed to provide empirical evidence of the influence of P-E interactions on the informal status and its relationship with taking charge. The situational factors examined in previous studies have been limited to organizational structures (Ibarra, 1993), racial–ethnic diversity (Sahib, 2015), narrative types, and values (Martin et al., 2016). Previous researchers have neglected the moderating effects of P-J and P-S fits in the fields of status and taking charge. This study thus extends the scope of application of P-E interaction factors to microlevel behaviors, thereby enriching the P-E fit theory. Moreover, this study also has theoretical implications regarding P-S fit. Based on interpersonal interaction theory, Glomb and Welsh (2005) discovered that the effects of P-S fit were extremely uncertain because the mechanisms of P-S fit were more complicated than previously imagined. Schaubroeck and Lam (2002) also suggested that the effect of P-S fit greatly depends on employees’ psychological characteristics. The empirical results showed that the knowledge workers’ informal status, OBSE, and P-E fit had played different roles in driving employees’ taking charge. Therefore, this study systematically uncovers the mechanisms by which P-J fit and P-S fit affect taking charge based on the self-consistency theory.

### Practical Implications

This study offers several insights for managers. First, organizations should pay more attention to P-J fit, because it can greatly improve employees’ willingness and ability to develop taking charge and therefore enhance organizational adaptability. The results show that P-J fit moderates the indirect effect of knowledge workers’ informal status on taking charge via OBSE, such that the indirect effect is strongest when the P-J fit is high. In other words, high P-J fit is beneficial to employees taking charge. Organizations should thus use effective selection processes to achieve P-J fit when hiring new members and managers should seek to enhance P-J fit, for instance, through selection and training, to improve employees’ taking charge.

Second, managers should make good use of P-S fit as an effective control tool to improve the organization’s overall initiative. The results show that P-S fit can enhance employees’ willingness and ability to take charge by strengthening their OBSE. Organizations should thus adopt the following methods to enhance social interactions and a sense of fit between leaders and subordinates. Teammates, supervisors, and subordinates’ personalities, values, and ways of doing things should all be taken into account. Teams with strong leadership and employees who are matched well in terms of values, personality, and behavior can reduce friction between supervisors and subordinates, avoid conflict, and greatly enhance effectiveness (Quade et al., 2017; Heyden et al., 2018). Organizations should minimize the distance between leaders and subordinates, for example, by increasing communication between supervisors and subordinates or conveying their personal views and behaviors to subordinates, which can help employees keep pace with their supervisors (Gardner et al., 2015). When selecting team leaders, organizations should prioritize employees compatible with existing members in terms of values, personality, and shared behavior.

Third, managers should emphasize employees’ OBSE to maximize the positive effects of spillover. Our results show that OBSE mediates the relationship between knowledge workers’ informal status and taking charge. Furthermore, the results show that P-J and P-S fits independently moderate the indirect effect of knowledge workers’ informal status on taking charge via OBSE, such that the indirect effect is strongest when they are high. Therefore, taking steps to improve members’ OBSE will be fundamental to enhancing an organization’s overall taking charge. OBSE is determined by individuals’ ability, influence, and respect; an organization that can improve employees’ sense of ability and value will therefore benefit. This is especially important for employees with low OBSE. Based on the notion of growth correlation (Liu et al., 2013), organizations should encourage managers to provide professional guidance for subordinates, such as skills training, mutual aid groups, or developing a mentorship system, to improve employees’ OBSE and enable the organization to harvest its beneficial spillover effects.

### Limitations and Future Research

This study has three limitations. First, although we collected data from multiple sources, data were collected at a single time point and the measure of taking charge was self-reported by employees. We recommend that future research collect multiwave data or use an experimental design to enhance the causality inference. Regarding the measure of taking charge, we suggest that future research adopt a measure based on the coworkers of focal employees.

Second, we had not examined the other potential mediators, for example, job autonomy and confidence. Previous research indicates that the higher-informal-status knowledge workers often have greater job autonomy than their colleagues (Polman et al., 2013), which can lead to higher rates of taking charge. Also, the higher-informal-status members will be more confident than their colleagues (Anderson et al., 2015), which promotes those employees to take charge more obviously. Therefore, future research can further empirically test the mediated effects of employees” job autonomy as well as confidence.

The third potential limitation is that we focused on the moderated effects of P-E fit and did not empirically examine other potential moderators, for instance, proactive personality and risk preference. These two moderators are likely to strengthen the positive relationship between knowledge workers’ informal status and taking charge. Therefore, future research could benefit from empirical tests of the moderating effects of proactive personality and risk preference.

## Data Availability Statement

The raw data supporting the conclusions of this article will be made available by the authors, without undue reservation, to any qualified researcher.

## Ethics Statement

Ethical review and approval was not required for the study on human participants in accordance with the local legislation and institutional requirements. The patients/participants provided their written informed consent to participate in this study.

## Author Contributions

CD developed the research model, analyzed the data, and co-drafted the manuscript. SL collected the data and co-drafted the manuscript. ZL and YB edited the manuscript. YZ analyzed the data and edited the manuscript. All authors contributed to the article and approved the submitted version.

## Conflict of Interest

The authors declare that the research was conducted in the absence of any commercial or financial relationships that could be construed as a potential conflict of interest.
